# Concurrent exposure to a dectin-1 agonist suppresses the Th2 response to epicutaneously introduced antigen in mice

**DOI:** 10.1186/1423-0127-20-1

**Published:** 2013-01-03

**Authors:** Jing-Yi Lin, Jau-Shiuh Chen, Pei-Chun Chen, Ming-Hui Chung, Ching-Yi Liu, Shi-Chuen Miaw, Li-Fang Wang

**Affiliations:** 1 Department of Dermatology, Chang Gung Memorial Hospital, Chang Gung University College of Medicine, Taoyuan, Taiwan; 2 Department of Dermatology, National Taiwan University Hospital, No.7, Chung-Shan South Road, Taipei, Taiwan; 3 Department of Statistics and Informatics Science, Providence University, Taichung, Taiwan; 4 Graduate Institute of Immunology of National Taiwan University Hospital and National Taiwan University College of Medicine, Taipei, Taiwan

**Keywords:** Fungus, Dectin-1, Epicutaneous sensitization, Th2 response

## Abstract

**Background:**

Epicutaneous sensitization with protein allergen that induces predominant Th2 responses is an important sensitization route in atopic dermatitis. Fungal components have been shown to modulate Th cell differentiation. However, the effects of fungal components on epicutaneous sensitization are unclear.

**Results:**

In this study, we showed that co-administration of curdlan, a dectin-1 agonist, during epicutaneous ovalbumin sensitization of BALB/c mice decreased the IL-5 and IL-13 levels in supernatants of lymph node cell ovalbumin reactivation cultures. Mechanistically, curdlan co-administration decreased IL-4 and IL-1β expressions in draining lymph nodes. Curdlan co-administration also lower the migration of langerin^+^ CD103^-^ epidermal Langerhans cells into draining lymph nodes at 96 hours post-sensitization which might be attributed to decreased expressions of IL-18 and IL-1β in patched skin. Moreover, adoptive transfer of CFSE-labeled transgenic CD4 T cells confirmed that curdlan co-administration decreased the proliferation and IL-4-production of ovalbumin -specific T cells primed by epidermal Langerhans cells.

**Conclusions:**

These results indicated that concurrent exposure to a dectin-1 agonist suppresses the epicutaneously induced Th2 response by modulating the cytokine expression profiles in draining LNs and the migration of epidermal Langerhans cells. These results highlight the effects of fungal components on epicutaneous allergen sensitization in atopic diseases.

## Background

The prevalence of atopic diseases has progressively increased in recent decades. The interaction between genetic susceptibility for atopy with varying environmental allergen exposures plays a central role in the pathogenesis of atopic diseases [1]. Allergens that provoke atopic diseases are ubiquitously distributed environmental protein antigens. Atopic dermatitis (AD) is often the first manifestation of the atopic triad and typically marks the onset of the “atopic march” [2].

The route of protein allergen sensitization in AD remains unclear. However, compelling clinical evidence suggests that epicutaneous exposure to protein antigen is one of the important sensitization routes for AD [3,4]. In animal models, we and others have demonstrated that epicutaneous sensitization with protein antigens induces predominate Th2 and weak Th1 responses, which leads to AD-like skin lesions and the development of asthma [5,6]. Epicutaneous sensitization with protein antigen also induces a modest Th17 response [7,8]. However, cross-priming with an epicutaneously introduced protein antigen generates Th1, but not Th2 cells [9]. An epicutaneously induced Th2 response requires the production of IL-10 and IL-13 [10,11]. Because defective IFN-γ production during infancy may be an important cause for sustained elevation of Th2 responses in atopic children, determining how to suppress Th2 and/or promote Th1 responses during the early sensitization period was expected to be a useful strategy to modulate the natural course of atopic diseases [12].

Severe systemic fungal infections have become an increasing problem during recent decades. The cell walls of fungi are composed, primarily, of carbohydrates, including mannoprotein, β-glucan, and chitin, which can be recognized by several classes of pattern recognition receptors [13]. Among these, dectin-1, a C-type lectin receptor involved in the recognition of β-glucan was shown to be crucial for the control of fungal infection [14,15]. Dectin-1 signaling in macrophages and neutrophils can trigger phagocytosis, a respiratory burst, and the production of inflammatory cytokines and chemokines, which further activate macrophages and neutrophils, thus resulting in the elimination of microorganisms [16]. Moreover, these pattern recognition receptors can drive the development of adaptive immunity. For example, signaling through dectin-1 induces dendritic cell (DC) maturation with the concomitant upregulation of co-stimulatory molecules and the secretion of IL-2, IL-10, IL-6, and TNF-α, in addition to a bias for IL-23 production rather than IL-12 [17]. Dectin-1-activated DCs can instruct the differentiation of Th1 and Th17, but not Th2 cells [17,18]. Stimulation of DCs via the dectin-1 pathway also allows priming of cytotoxic T-cell responses [19].

Many fungi, such as *Candida albicans*, are both commensals and pathogens at the skin surface and mucosa. In addition, cutaneous superficial dermatophyte infections are very common in subtropical and tropical regions. Thus, the effects of concurrent exposure to fungal components on epicutaneous sensitization with protein antigen need to be explored. Curdlan is a pure (1–3) β-glucan in triple helix form that can specifically activate dectin-1 signaling [17]. In this study, we demonstrate that concurrent curdlan exposure can suppress the predominate Th2 response induced by epicutaneous sensitization with protein antigen.

## Methods

### Mice and reagents

Eight to 12-week-old female BALB/c mice were purchased from the animal center of National Taiwan University College of Medicine and maintained in a specific pathogen-free environment. All animal experiments were approved by the animal care committee of the Medical Collage of National Taiwan University. Ovalbumin (OVA) (Grade V) was purchased from Sigma-Aldrich (St Louis, Mo) and curdlan was from Wako (Richmond, VA). Pam3CSK4 and zymosan were from InvivoGen (Carlsbad, CA). Capture and biotin-conjugated detection antibodies against IFN-γ and IL-5 used for ELISAs were from PharMingen (San Diego, CA). Streptavidin-alkaline phosphatase was from Southern Biotechnology (Birmingham, AL). A murine IL-13 ELISA kit (R&D Systems, Minneapolis, MN) and IL-17 ELISA kit (eBioscience) were used for determinations of IL-13 and IL-17 in cell culture supernatants. Antibodies for flow cytometry were from PharMingen, e-Bioscience (San Diego, CA) or Miltenyi (Bergisch Gladbach, Germany). Antibody-conjugated microbeads used for isolating cells were from Miltenyi (Bergisch Gladbach, Germany).

### Epicutaneous sensitization

Mice were sensitized as previously described [9]. Briefly, 20 μL of OVA (100 mg/mL) was placed on the disc of a Finn chamber (Epitest, Finland). This disk was applied to an area of shaved skin on the back of a mouse. During the sensitization course, freshly prepared patches were applied daily on days 1–5. For groups of mice that received co-administration of curdlan, 10 μL of 25 μg/μl or 50 μg/μl curdlan suspensions were added to each Finn chamber disc. For groups of mice that received co-administration of Pam3CSK4 or zymosan, 10 μL of Pam3CSK4 (0.5 μg/μL) or zymosan (2 μg/μL) were added.

### Cytokines in supernatants of reactivation cultures

Ten days after beginning the sensitization course, mice were sacrificed and axillary, subscapular, and inguinal lymph nodes (LNs) were harvested. Pooled LN cells (1×10^6^) were cultured in the presence or absence of 100 μg/mL OVA. Supernatants were harvested 48 h later and stored at −80°C. IFN-γ, IL-5, IL-13, and IL-17 in supernatants were measured by standard sandwich ELISAs. Detection limits for IL-5, IL-13, and IL-17 were 10 pg/mL, and the limit for IFN-γ was 50 pg/mL. OVA-specific cytokine production was calculated by subtracting cytokine production measured in the absence of OVA from that in the presence of OVA.

### Flow cytometric analysis of DC subsets in draining LNs

Skin-draining LNs were excised at 24 or 96 hours after the start of the sensitization course. LN cell suspensions were prepared by digestion with 2.5 mg/ml collagenase for 30 min at 37°C, the tissue was ground, then resuspended in HBSS containing 10 mM EDTA for another 5 minutes. CD11c^+^ cells were isolated using anti-CD11c microbeads. Cells were stained using various combinations of the following antibodies: CD11c-APC (HL3), CD103-FITC (2E7), mPDCA-1-PE (JF 05-1C 2.4.1), B220-biotin (RA3-6B2), MHC class II-PE-cy5 (2 G9), CD8-biotin (53–6.7), CD40-biotin (3/23), CD80-biotin (16-10A1), CD86-biotin (PO3), CD24-biotin (M1/69) and their isotype controls with or without subsequent staining with streptavidin-APC or streptavidine-PE. Intracellular staining for anti-langerin (CD207)-PE was also done.

### Total RNA extraction, cDNA preparation and quantitative real-time PCR

Skin was obtained at 2, 4, and 24 hours after patch application. Draining LNs were obtained at 24, 48, 72, and 96 hours after patch application. Primary keratinocytes were obtained 0.5, 2, 4 and 24 hours after *in vitro* culture. These were frozen with liquid nitrogen and soaked in 1 ml TRIzol Reagent (Invitrogen, CA,). After homogenization, total RNA was extracted, cDNA was synthesized and the expressions of mRNA for IFN-γ, IL-4, IL-6, IL-10, IL-12 (p35), IL-12 (p40), IL-18, IL-23, TGF-β, IL-1α, IL-1β, and TNF-α were determined by quantitative real-time PCR according to the manufacturer’s instructions. Each sample was analyzed in duplicate. Relative cytokine mRNA expression levels were normalized to β-actin expressions.

### Adoptive transfer

For adoptive transfer of OVA-TCR CD4 T cells, spleen cells from DO.11.10 mice were positively selected for CD4 T cells using CD4 microbeads. Then, 10^7^ CD4 T cells/ml were incubated with 1 μM CFSE for 10 min at 37°C. Pre-warmed FCS-containing PBS was added and then washed with cold PBS. Labeled OVA-TCR CD4 T cells (5×10^6^) were intravenously injected into BALB/c recipients 3 days after the start of the sensitization course. Draining LNs were harvested 3 days after transfer. Flow cytometric analysis was used after surface staining for CD4 or intracellular staining for IL-4.

## Results

### Co-administration of a dectin-1 agonist suppresses the predominant Th2 response induced by epicutaneous sensitization with protein antigen

Curdlan is a (1–3) β-glucan that specifically activates dectin-1 signaling. To examine the effects of a dectin-1 agonist on epicutaneous sensitization with protein antigen, we used a well-established murine protein-patch model and co-administered doses of curdlan along with OVA by patch application. Ten days after beginning the sensitization course, LN cell OVA reactivation cultures were performed and cytokine contents in supernatants were measured to determine the direction and amplitude of induced OVA-specific Th responses.

**Figure 1:**
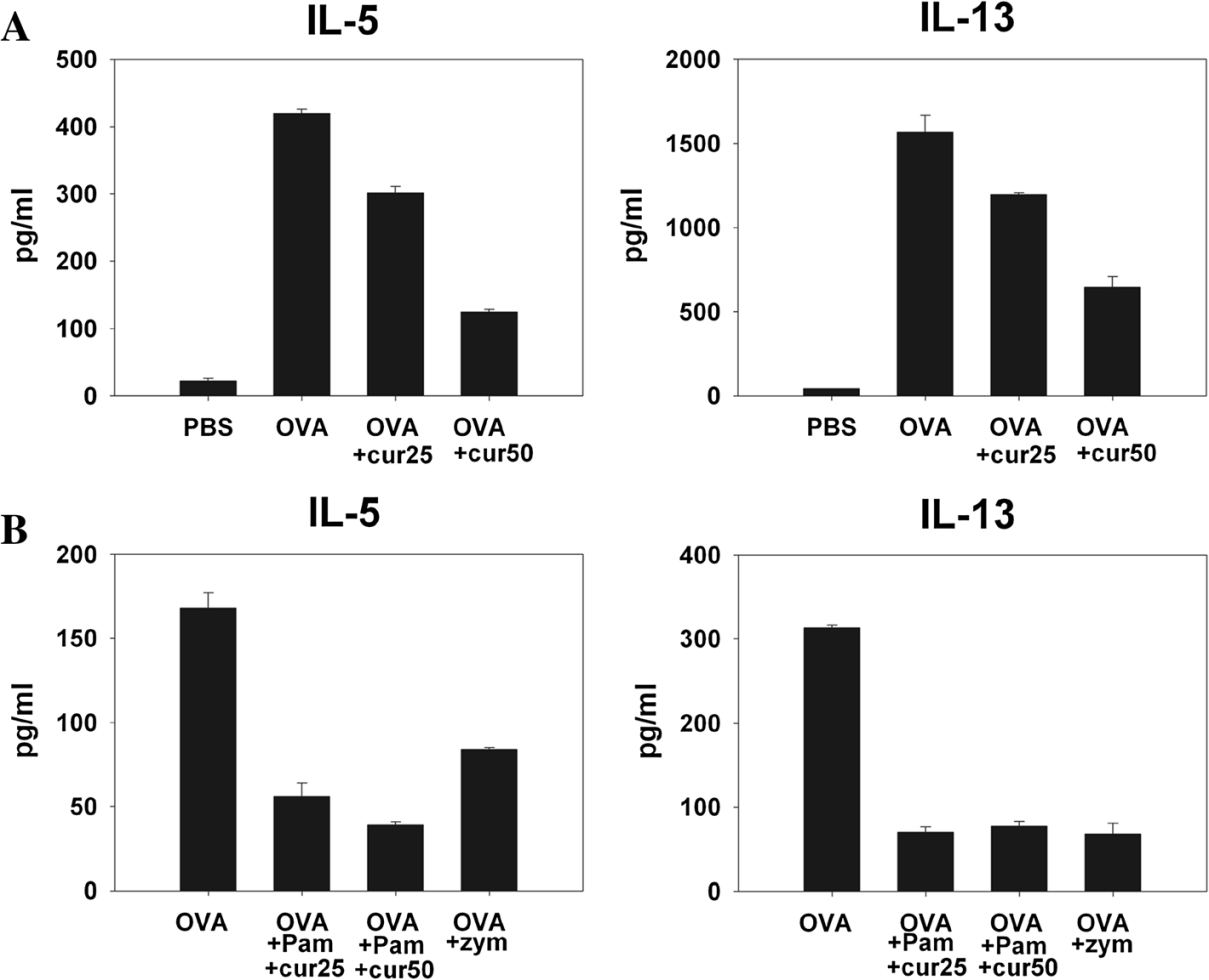
**Co-administration of dectin-1 agonists suppresses the epicutaneously OVA-induced Th2 immune response. (A)** Groups of BALB/c mice (n=5) were sensitized by patch application with OVA plus doses of curdlan or OVA alone on day1-5. **(B)** Groups of BALB/c mice (n=5) were sensitized by patch application with OVA plus zymosan, OVA plus Pam3CSK4 and doses of curdlan, or OVA alone on day 1–5. Ten days after the start of the sensitization course, draining LNs were obtained. The IL-5 and IL-13 contents in supernatants of in vitro OVA reactivation culture of LN cells were determined by ELISA. Net concentrations (concentration in the absence of OVA subtracted from concentration in the presence of OVA) were presented. Data are representative of at least three independent experiments.

As expected, compared with the PBS control group, mice that received OVA patch applications showed markedly higher IL-5 and IL-13 levels in culture supernatants (Figure 1A). Two groups of mice that received co-administration of varying doses of curdlan along with OVA showed lower IL-5 and IL-13 levels in culture supernatants compared to mice that received OVA alone (Figure 1A). The IFN-γ and IL-17 levels in supernatants from groups of mice that received OVA plus curdlan varied (data not shown). They were mostly lower than mice that received OVA alone. However, higher IFN-γ and IL-17 levels than mice receiving OVA alone were occasionally detected. Nevertheless, lower IL-5 and IL-13 levels in supernatants compared to mice receiving OVA alone were always detected despite variation of IFN-γ and IL-17 levels. Mice that received curdlan alone without OVA had levels of IFN-γ, IL-5, IL-13, and IL-17 in supernatants similar to PBS controls (data not shown).

Zymosan is a mixture of (1–3) and (1–6) β-glucans and is a stimulus for both dectin-1 and TLR2 [20]. The importance of the collaboration between these 2 receptors for regulating induced Th responses has been emphasized [20]. Therefore, we checked the effects of co-administering Pam3CSK4 (TLR2 agonist) plus curdlan (dectin-1 agonist), as well as zymosan, on epicutaneous sensitization with OVA. The effect of Pam3CSK4 alone on OVA epicutaneous sensitization has been tested in our previous study, co-administration of Pam3CSK4 with OVA showed a modest increase of OVA-specific Th2 response when compared with patch application with OVA alone. As shown in Figure 1B, compared to mice that received OVA alone, the groups of mice that were co-administered Pam3CSK4 plus doses of curdlan along with OVA had lower IL-5 and IL-13 levels in the supernatants of LN cell OVA reactivation cultures. Mice that were co-administered zymosan with OVA also had lower IL-5 and IL-13 levels than the mice that received OVA alone. The levels of IFN-γ and IL-17 in the supernatants for these groups of mice were again variable compared to mice that received OVA alone (data not shown). Taken together, these results indicate that co-administration of a dectin-1 agonist, including curdlan and zymosan, suppresses the predominant Th2 response induced by epicutaneous sensitization with protein antigen.

### Co-administration of a dectin-1 agonist decreases the expression levels of IL-4 and IL-1β in draining LNs

**Figure 2:**
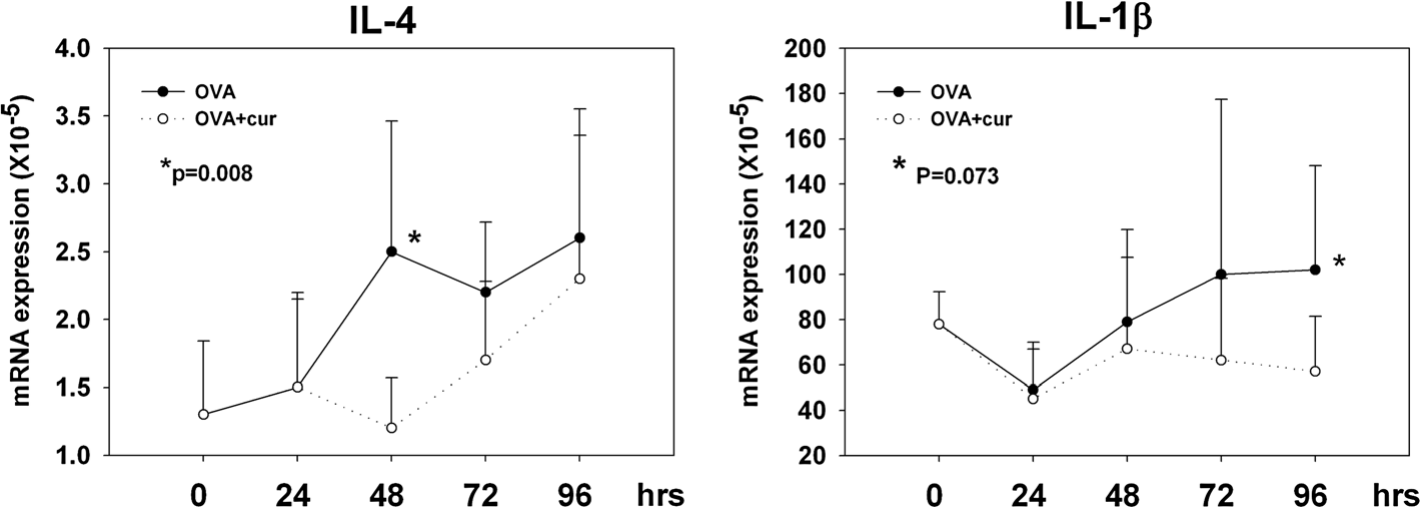
**Co-administration of curdlan with OVA influences the cytokine expression profiles in draining LNs.** Groups of BALB/c mice were sensitized by patch application with OVA plus curdlan or OVA alone. Draining LNs were obtained at 24, 48, 72 and 96 hours after sensitization. Total RNA extraction, cDNA preparation and quantitative real-time PCR for various cytokines were performed. The relative cytokine mRNA expression of each group was normalized to its β-actin expression. Results are shown as means and standard deviations of pooled data from five independent experiments. P values was calculated by Wilcoxon Rank Sum test.

Cytokine expression patterns in the microenvironmental milieu at sites of T cell priming orchestrate the direction and amplitude of induced Th responses. Thus, in order to explore the underlying mechanisms of suppressive effect of curdlan co-administration, we sought to analyze the cytokine expression profiles in draining LNs at 24, 48, 72, and 96 hours after epicutaneous sensitization. IFN-γ, as well as IL-12, and IL-4 are generally regarded as the key cytokines that direct Th1 and Th2 responses, respectively, whereas IL-6 and TGF-β are involved in Th17 differentiation. IL-18 is unique in that it can promote either Th1 or Th2 differentiation, depending on the presence or absence of IL-12. IL-10 and IL-1β may be particularly critical for epicutaneously-induced immune responses [11]. In addition, we checked the mRNA expression levels of pro-inflammatory cytokines, including IL-1α and TNF-α. As shown in Figure 2, the mRNA expression levels of IL-4 in draining LNs increased gradually after the start of epicutaneous sensitization with OVA. The IL-4 expression levels of mice that received OVA plus curdlan were notably lower than those of mice that received OVA alone. The difference reached statistical significance at 48 hr timepoint (p=0.008). Mice that received OVA plus curdlan also showed a trend of lower IL-1β expression levels than mice that received OVA alone, but the differences only approached statistical significance at 96 hr timepoint (p=0.073). The expression levels of IFN-γ, IL-12 (p35), IL-12 (p40), IL-6, TGF-β, IL-18, IL-1α, and TNF-α in draining LNs at 24, 48, 72, and 96 hours post-sensitization were similar between these 2 groups (data not shown). Thus, co-administering curdlan during epicutaneous OVA sensitization decreased the IL-4 and IL-1β expressions in draining LNs, which might contribute to its suppressive effect on induced Th2 response.

### Co-administration of a dectin-1 agonist decreases the migration of epidermal Langerhans cell into draining LNs

The migration and activation of DCs also play a central role in T cell priming. Therefore, we analyzed the numbers and characteristics of skin-derived migratory DCs in draining LNs. Skin-derived migratory DCs express the highest levels of MHC class II molecules in draining LNs and are divided into 3 subsets: epidermal Langerhans cells (LC; langerin^+^ CD103^-^) , dermal langerin^+^ DCs (langerin^+^ CD103^+^), and dermal langerin^-^ DCs. Dermal langerin^+^ DCs arrive at draining LNs significantly earlier than epidermal LCs, with peak influxes at 24 and 96 hours post-epicutaneous sensitization, respectively [21]. We did not check dermal langerin^-^ DCs, as there are no trustworthy markers for this subset. Blood-derived, LN-resident DC subsets, including CD8^+^ DCs (CD8^+^ MHC class II ^medium^) and plasmacytoid DCs (mPDCA-1^+^ B220^+^), were also analyzed as references.

**Figure 3:**
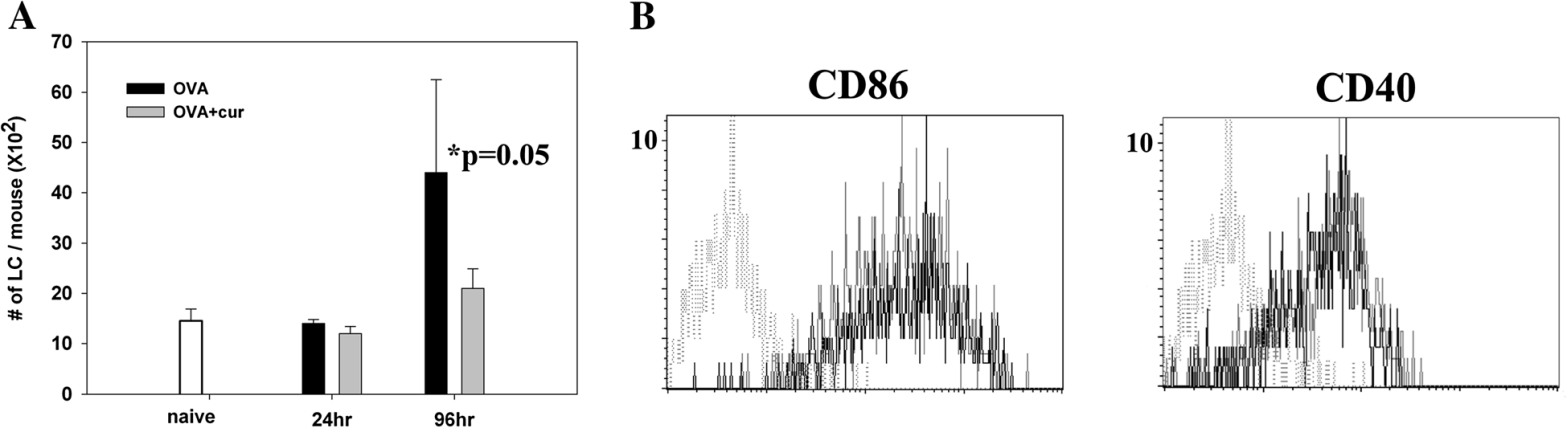
**Co-administration of curdlan decreases the number of epidermal LC in draining LNs at 96 hours post-epicutaneous sensitization. (A)** Groups of BALB/c mice were sensitized by patch application with OVA plus curdlan or OVA alone. 24 and 96 hours after patch application, draining LNs were obtained and prepared for positive selection for DCs. Intracellular staining for langerin and surface staining for CD103 were performed. Langerin^+^ CD103^-^ cells represent epidermal LCs. The results were shown as mean ± SD of pooled data from four independent experiments. **(B)** 96 hours after patch application, intracellular staining for langerin and surface staining for CD103 and various costimulatory molecules were performed. Langerin^+^ CD103^-^ cells were gated. Expression levels of CD86 and CD40 were shown. OVA group (gray line), OVA plus curdlan group (black line), isotype control (gray dotted line).

The total numbers of CD11c^+^ DCs in draining LNs at 24 or 96 hours after epicutaneous sensitization were similar between mice that received OVA alone or mice that received OVA plus curdlan (data not shown). The numbers of epidermal LC (langerin^+^ CD103^-^) in draining LNs were similar between these 2 groups at 24 hours after epicutaneous sensitization. However, at 96 hours after epicutaneous sensitization, the numbers of epidermal LCs of mice that received OVA plus curdlan were significantly lower than for mice that received OVA alone (p=0.05) (Figure 3A). The numbers of dermal langerin^+^ DC (langerin^+^ CD103^+^), CD8^+^ DC (CD8^+^ MHC class II ^medium^), plasmacytoid DC (mPDCA-1^+^ B220^+^) were similar between the 2 groups of mice at 24 and 96 hours after epicutaneous sensitization (data not shown).

We next checked the expressions of co-stimulatory molecules on langerin^+^ CD103^-^ epidermal LCs in draining LNs at 96 hours after epicutaneous sensitization. As shown in Figure 3B, the expression levels of CD86 and CD40 were comparable between the 2 groups of mice. The expression levels of CD80 and CD24 were also comparable. Taken together, these results indicate that co-administering a dectin-1 agonist during epicutaneous sensitization with protein antigen results in decreased migration of epidermal LCs into draining LNs without modulating their expressions of co-stimulators.

### Co-administration of a dectin-1 agonist decreases the expression levels of IL-18 and IL-1β in patched skin

**Figure 4:**
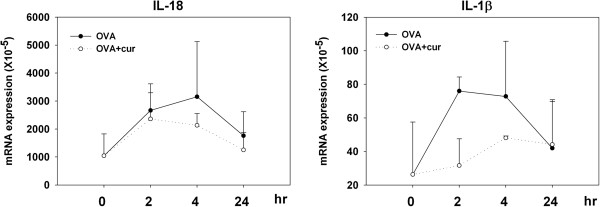
**Co-administration of curdlan with OVA influences the cytokine expression profiles in patched skin.** Groups of BALB/c mice were sensitized by patch application with OVA plus curdlan or OVA alone. Patched skins were excised 2, 4 and 24 hours after patch application. Total RNA extract, cDNA preparation and quantitative real-time PCR for various cytokines were performed. The relative cytokine mRNA expression of each group was normalized to its β-actin expression. Results are shown as means and standard deviations of pooled data from four independent experiments.

**Figure 5:**
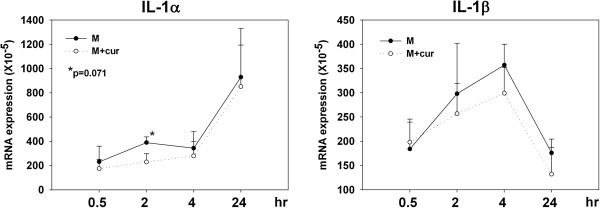
**Presence of curdlan in*in vitro*culture decreases the IL-1α and IL-1β expressions of primary keratinocytes.** Skin was obtained from naïve BALB/c mice and prepared into epidermal cell suspension. Keratinocytes were purified by negative selection with MHC class II bead. In vitro culture of primary keratinocytes in the absence or presence of curdlan (50 μg/ml) were performed. Keratinocytes were harvested 0.5, 2, 4 and 24 hours after the start of culture. Measurement of various cytokines were performed similar to Figure 4.

It has been reported that IL-18 induces LC migration by a TNF-α and IL-1β-dependent mechanism [22,23]. Thus, to explore the underlying mechanism decreasing the migration of epidermal LCs, we next checked the effect of curdlan co-administration with OVA on the expression levels of these cytokines in patched skin. As shown in Figure 4, the mRNA expression levels of IL-18 and IL-1β had already increased 2 hours after OVA patch application. Mice that received OVA plus curdlan showed a trend of lower expression levels of IL-18 in patched skin than mice that received OVA alone at 2, 4, and 24 hours after patch application. Mice that received OVA plus curdlan also showed a trend of lower expression levels of IL-1β at 2 and 4 hours after patch application. However, the differences didn’t reach statistical significance. The expression levels of TNF-α as well as IL-1α, IL-6 and TGF-β in patched skin showed no significant differences between the 2 groups of mice (data not shown). To further elucidate the target cell of the effects of curdlan in patched skin, primary keratinocytes purified from the epidermis of naïve BALB/c mice were cultured in the absence or presence of curdlan for 24 hours. The cytokine expression levels were measured at 0.5, 2, 4, and 24 hours after the start of culture. Figure 5 shows that the expression levels of IL-1β as well as IL-1α increased after the start of keratinocyte culture. The presence of curdlan in the culture media seemed modestly decreasing the expression levels of IL-1β and IL-1α. However, only the difference of expression level of IL-1α at 2 hr timepoint approached statistical significance (p=0.071). In contrast, the expression levels of IL-18 and TNF-α, IL-6 and TGF-β decreased gradually after the start of keratinocyte culture and their expression levels were comparable in the absence or presence of curdlan (data not shown). Collectively, these results showed that curdlan co-administration modulated the cytokine expression levels of patched skin, that might contribute to its decreasing effect on migration of epidermal LCs.

### Co-administration of curdlan decreases the proliferation and IL-4 production of specific T cells primed by migratory epidermal LCs in draining LNs

**Figure 6:**
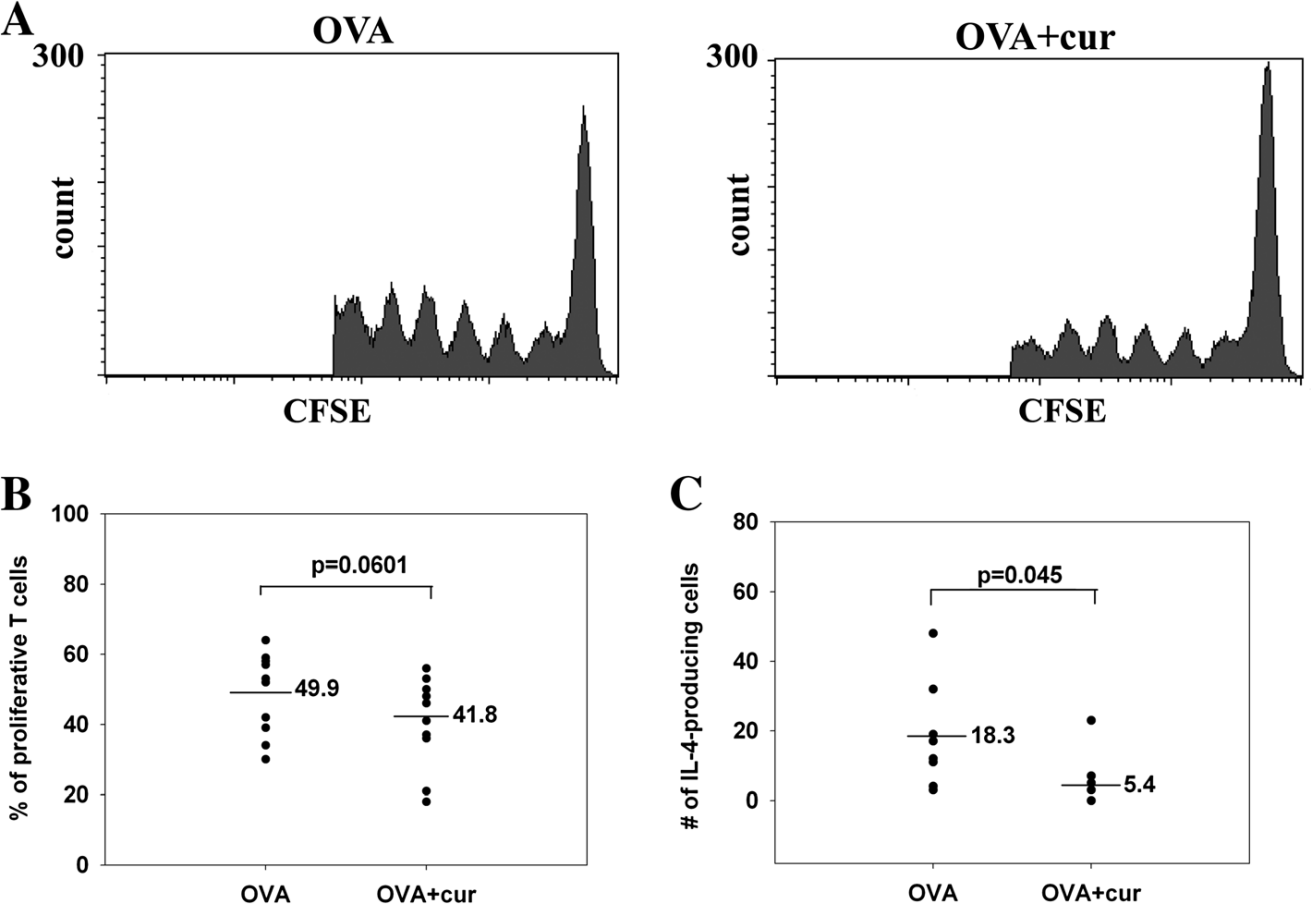
**Co-administration of curdlan decreases the proliferation and IL-4 production of specific T cells primed by epidermal LCs.** CFSE-labeled CD4 T cell from DO.11.10 mice were intravenously transferred into groups of BALB/c mice three days after they received patch application with OVA plus curdlan or OVA alone. Draining LNs were obtained three days after transfer. **(A)** After staining with CD4, flow cytometric analysis was performed. CD4+ CFSE+ cells were gated. **(B)** Pooled results of percentages of divided, labeled CD4 T cells of individual mouse from three independent experiments (n=12) were shown. **(C)** With intracellular staining for IL-4, pooled results of numbers of IL-4-producing, labeled CD4 T cells of individual mouse from two independent experiments were shown (n=8).

To further explore the effects of curdlan co-administration on epidermal LCs *in vivo*, CFSE-labeled OVA-specific TCR-transgenic CD4 T cells from DO11.10 mice were adoptively transferred into BALB/c mice at 3 days after beginning the OVA sensitization course, a time point when epidermal LCs arrived at draining LNs. The draining LNs were harvested 3 days after the transfer. The division of CFSE-labeled T cells in draining LNs was analyzed by flow cytometry. Figure 6A shows that CFSE-labeled CD4 T cells divided rapidly in draining LNs. Notably, mice that were co-administered curdlan showed a significantly lower percentage of divided labeled CD4 T cells compared to mice that received OVA alone (Figure 6B). The difference approached statistical significance (p=0.06). Moreover, intracellular IL-4 staining showed that mice that were co-administered curdlan had fewer IL-4-producing labeled CD4 T cells than mice that received OVA alone (p=0.045; Figure 6C). In contrast, when CFSE-labeled OVA-specific TCR-transgenic CD4 T cells were adoptively transferred 1 day before the start of the sensitization course, mice that received OVA plus curdlan had a comparable percentage of divided labeled CD4 T cells to that of mice that received OVA alone (data not shown). Thus, co-administration of curdlan decreased the proliferation and IL-4 production of specific T cells primed by migratory epidermal LCs in draining LNs.

## Discussion

In this study, we demonstrated that concurrent exposure to a dectin-1 agonist could suppress the predominant Th2 response induced by epicutaneous sensitization with protein antigen. We also showed that co-administering a dectin-1 agonist influenced the cytokine expression profiles in draining LNs and decreased the migration of epidermal LCs. To our knowledge, the effects of fungal components on epicutaneous sensitization with protein antigen have not been previously reported.

The effect of dectin-1 signaling on the activation of DCs has been investigated using in vitro culture systems. Carmona et al. and LeibundGut-Landmaun et al. both reported that adding a dectin-1 agonist to DC culture activated the DCs, as reflected by increased surface expressions of CD80, CD86, and CD40 [17,18]. At first glance, our results showing a comparable expression of co-stimulators on LCs in draining LNs would seem to be in conflict with previous reports. However, dectin-1 was shown to be expressed on langerin^-^ dermal DCs, but not langerin^+^ epidermal LCs [24]. In addition, although epidermal keratinocytes do not constitutively express dectin-1, it can be induced by β-glucan [25]. Therefore, in our experimental system, epidermal LCs could not directly respond to co-administered curdlan. Instead, they were indirectly influenced by cytokines that were released by surrounding keratinocytes that responded to curdlan via their induced dectin-1. Moreover, IL-18 and IL-1β have been shown to govern epidermal LC migration from the epidermis into draining LNs [22,23]. Our results showing decreased expressions of IL-18 and IL-1β in the patched skin of mice that were co-administered curdlan with OVA are in agreement with the decreased migration of epidermal LCs into draining LNs.

Among all of the cytokines analyzed, IL-18 is always the most abundantly expressed in skin. IL-18, a member of the IL-1 family, is produced as a biologically inactive precursor and becomes active after its cleavage by caspase-1 [26]. Keratinocytes constitutively express IL-18 and the IL-18 receptor [27]. The expression levels of IL-18 by keratinocytes are up-regulated when exposed to double-stranded RNA, β-defensin, and cathelicidin, whereas its expression is suppressed by vitamin D3 [28-30]. Functionally, IL-18 can induce CXCL10 production by keratinocytes, IL-4 and IL-13 secretion by mast cells and basophils, and the migration of epidermal LCs [24,31,32]. Moreover, AD-like inflammatory skin lesions develop spontaneously in transgenic mice that overexpress mature IL-18 in their skin [33]. Single nucleotide polymorphisms of the IL-18 gene have been shown to be associated with AD [34]. Our results that showed decreased IL-18 expressions in patched skin and decreased migration of epidermal LCs into draining LNs when curdlan was co-administered with OVA are in agreement with a lower level of induced Th2 response. Moreover, our results that showed no significant differences of IL-18 expressions in the draining LNs between OVA and OVA plus curdlan groups support the notion that IL-18 does not play a critical role at the priming site during epicutaneous sensitization with protein antigen.

The expression level of IL-1β by epidermal keratinocytes is one of the decisive factors for generating protective Th1 immunity during experimental Leishmaniasis, along with IL-12, IL-4, IL-6, and osteopontin [35]. After OVA patch application, the mRNA expression levels of IL-1α, IL-1β, IL-6, IL-18, and TGF-β of patched skin increased rapidly when compared with naïve BALB/c mice, whereas the expressions of TNF-α and IL-23 were at levels similar to naïve mice (data not shown). We could not detect any significant expressions of IFN-γ, IL-4, IL-10, IL-12 (p35), or IL-12 (p40) in patched skin. The lack of IL-4 and IL-12 expressions in patched skin might contribute to the predominant Th2 responses. The significance of the induced expressions of IL-1α, IL-1β, and IL-6 in OVA-patched skin is obscure at present. However, we suggest that they function as potentiators and enhancers of cutaneous inflammatory responses, similar to IL-18.

The effects of fungal components on adaptive immunity has been investigated using in vivo systems. Dillon et al. reported that zymosan induced regulatory APCs and immunological tolerance in an experimental mouse system using immunization by intravenous injections of zymosan plus OVA [36]. Karumuthil-Melethil et al. reported that treating pre- and early hyperglycemic NOD mice with zymosan resulted in suppressing insulitis, leading to a significant delay in hyperglycemia [37]. Both reports showed that zymosan treatment-induced suppression was associated with enhanced suppressor functions of CD4^+^CD25^+^ T regulatory cells, which produced large amounts of IL-10 upon activation. Our results showing suppression of a predominant Th2 response are in agreement with these observations, although we did not detect large amounts of IL-10 in LN cell or splenocyte reactivation cultures (data not shown). In contrast, Yoshitomi et al. reported that a single intraperitoneal injection of zymosan could trigger severe chronic arthritis in SKG mice, which failed to develop the disease in microbially clean conditions [38]. Moreover, Kobayashi et al. reported that an asthma-related environmental fungus, *Alternaria*, produced potent Th2 adjuvant effects in the airways [39]. Possible reasons for the remarkable discrepancies between this report and our results are the following. First, Th2 skewing is stronger and absolute with epicutaneous sensitization, while it is only preferential with inhalation priming, as evidenced by detectable antigen-specific IgE in only a minority of inhalation-sensitized mice and that some mice also exhibited antigen-specific IgG2a responses [30]. Second, marked differences in the IL-4 and IL-13 dependencies of Th2 responses generated on the skin or in the airways have been emphasized [40]. Third, the study by Kobayashi et al. used fungal extracts that may have induced signaling other than dectin-1. Fourth, during inhalation sensitization, fungal elements can be approached by dectin-1 expressing macrophages and DCs in the alveoli, which is quite a different situation from non-dectin-1-expressing epidermal LCs in the skin.

## Conclusion

Concurrent exposure to a dectin-1 agonist could suppress the predominant Th2 immune response induced by epicutaneous sensitization with a protein antigen by decreasing the migration of epidermal LCs and influencing the cytokine profiles of draining LNs. These results provide useful information for the prevention and control of allergen sensitization in atopic diseases.

## Abbreviations

AD: Atopic dermatitis; DC: Dendritic cell; OVA: Ovalbumin; LN: Lymph node.

## Competing interests

The authors declare that they have no competing interests.

## Authors’ contributions

JYL, JSC and LFW participated in the design of the study and preparation of the manuscript. MHC and CYL performed all experiments. PCC performed the statistical analysis. All authors read and approved the final manuscript.
